# Identification and Characterization of a Novel Luteovirus Infecting *Hosta ventricosa* Plants

**DOI:** 10.3390/v18070798

**Published:** 2026-07-20

**Authors:** Liyan Li, Lele Chen, Tongkun Guo, Li Xie, Shuai Fu, Jianxiang Wu

**Affiliations:** 1 State Key Laboratory of Rice Biology and Breeding, Zhejiang Key Laboratory of Biology and Ecological Regulation of Crop Pathogens and Insects, Institute of Biotechnology, Zhejiang University, Hangzhou 310058, China; 12016091@zju.edu.cn (L.L.); 22360088@zju.edu.cn (L.C.); 2 Hainan Institute of Zhejiang University, Sanya 572025, China; 3 Research Center for Life Sciences Computing, Zhejiang Lab, Hangzhou 311100, China; guotanken@gmail.com; 4 Analysis Center of Agrobiology and Environmental Sciences, Zhejiang University, Hangzhou 310058, China; hyena_xieli@163.com

**Keywords:** novel virus, *Hosta ventricosa*, luteovirus, viral infectious clone, viral pathogenicity determinant, RNA silencing suppressor

## Abstract

*Hosta ventricosa*, also known as blue plantain lily, is an important traditional herbal medicinal and ornamental plant in China. Prior to this study, no virus has been reported to infect *H. ventricosa* plants. Based on RNA-seq, transmission electron microscopy, and RT-PCR analyses, we have demonstrated that the *H. ventricosa* plant showing leaf chlorosis, mottle, mosaic, and crinkling symptoms was co-infected with hosta virus X (HVX) and a novel luteovirus, which we tentatively named hosta ventricosa luteovirus (HVLV). The genome of HVLV is a 5723 nt long, positive-sense, and single-stranded RNA with seven open reading frames (ORFs). Phylogenetic analysis based on the amino acid (aa) sequence of the viral RNA-dependent RNA polymerase (RdRp) revealed that HVLV is clustered within the genus *Luteovirus*. The HVLV RdRp shares 9.75–45.17% aa sequence identity with the 14 closely related luteoviruses. The P1–2 and P3–5 proteins of HVLV were identified as potential viral pathogenicity determinants through the PVX heterologous expression in *Nicotiana benthamiana* plants. Moreover, P2, P5 and P3–5 proteins of HVLV have been found to exhibit RNA silencing suppression activities. Additionally, we have successfully constructed an infectious cDNA clone of HVLV and uncovered that this infectious cDNA clone can infect *N. benthamiana* plants through agroinfiltration. These findings have expanded our understanding of luteoviruses and their host range.

## 1. Introduction

*Hosta ventricosa*, also known as blue plantain lily, is a perennial herbaceous plant in the genus Hosta, within the family Liliaceae. *H. ventricosa* is one of the four endemic species commonly cultivated in China as landscaping and ornamental plants [1]. In addition, *H. ventricosa* can be used as a herbal medicine plant with anti-inflammatory and anti-tumor activities [2,3]. To date, hosta virus X (HVX), a potexvirus, has been reported to infect various species within the Hosta genus and the HVX-infected plants often show leaf mosaic, interveinal chlorosis, and leaf desiccation. However, its infection does not produce obvious symptoms in more tolerant cultivars [4].

Luteoviruses are a group of positive-sense single-stranded RNA (+ssRNA) viruses in the genus *Luteovirus*, the family *Tombusviridae* [5]. All members in the genus *Luteovirus* possess spherical virions of 25–30 nm in diameter. Luteoviruses are known to only infect phloem cells in host plants and are transmitted by aphids in a circulative and non-propagative manner [6]. Luteovirus genomes are 5.3 kb to 5.9 kb in length and contain six major open reading frames (ORFs). The ORF1 and ORF2 encode two replication-related proteins [6]. The ORF2 is translated from the nucleotide position 1269 via a −1 translational frameshift mechanism to produce the viral RNA-dependent RNA polymerase (RdRp) [7]. The ORF3 encodes the viral capsid protein (CP). ORF5 is translated via readthrough of the ORF3 stop codon to generate an ORF3-ORF5 fusion protein. The ORF5-encoded readthrough domain (RTD) is essential for aphid transmission [8]. The ORF3a encodes a protein responsible for virus long-distance movement [9], and the ORF4 encodes a cell-to-cell movement protein (MP) [8]. Notably, it was reported that ORF4 is absent or non-functional in some luteoviruses [10,11,12]. ORF6, present only in certain luteoviruses, encodes an uncharacterized small protein [11,12,13,14].

Identification of plant-infecting viruses is crucial for sustainable agriculture and landscaping. Common methods currently used to identify known plant RNA viruses mainly include serological assays (e.g., enzyme-linked immunosorbent assay) and reverse transcription-polymerase chain reaction (RT-PCR). However, these commonly used methods are inadequate for the identification of previously uncharacterized plant viruses [15]. Notably, high-throughput RNA sequencing (RNA-seq) is a relatively new technology and is very powerful for novel virus identification [10,16,17,18,19,20,21,22,23].

In this study, we aimed to identify the viral agent(s) responsible for the observed chlorosis, mottle, and crinkling symptoms in *H. ventricosa* plants and to characterize the biological properties of the encoded viral proteins.

## 2. Materials and Methods

### 2.1. Plant Materials and Growth Conditions

In 2021, a *H. ventricosa* plant showing leaf chlorosis, mottle, mosaic and crinkling symptoms was sampled from the China National Botanical Garden in Beijing, China. Multiple leaves were collected from this plant and used for RNA-seq. Seeds of the 16c transgenic *N. benthamiana* line widely used for RNA silencing suppression assays were kindly provided by Professor David C. Baulcombe (University of Cambridge, Cambridge, UK) [24,25]. The *H2B-RFP* transgenic *N. benthamiana* line expresses a fusion protein of histone H2B and red fluorescent protein (RFP), which labels the nuclei [26]. All assay plants were grown in a plant growth chamber maintained at 25 °C and 18 °C (day/night), with 60% relative humidity, and a 16 h light and 8 h dark photoperiod.

### 2.2. RNA Sequencing (RNA-Seq) and De Novo Assembly

Total RNA was extracted from the *H. ventricosa* plant sample using the EASYspin Plus Complex Plant RNA Kit (Aidlab Biotech, Beijing, China). Ribosomal RNAs were removed from the total RNA samples using the TruSeq RNA Sample Prep Kit (Illumina, San Diego, CA, USA) and the resulting RNA samples were used to construct RNA libraries using the TruSeq RNA Sample Prep Kit as instructed (Illumina, San Diego, CA, USA). RNA sequencing was performed on an Illumina HiSeq X-ten platform by Biomarker Technologies (Beijing, China). Low-quality reads were filtered and the adapters of the paired-end raw reads were trimmed using the CLC Genomics Workbench 9.5 software (QIAGEN, Hilden, Germany). All clean reads were de novo assembled into contigs using the Trinity v2.3.2 program (Broad Institute and the Hebrew University of Jerusalem, Cambridge, Massachusetts and Jerusalem, USA and Israel). To identify viral contigs, the resulting contigs were then searched against the NCBI non-redundant (nr) nucleotide and protein databases using BLASTn and BLASTx (version 2.16.0) (E-value ≤ 1 × 10^−5^). Contigs were classified as viral if they encoded conserved the viral RNA-dependent RNA polymerase (RdRp) or coat protein (CP) protein domains. Candidate viral contigs were further compared with known viral sequences in the GenBank database.

### 2.3. RT-PCR and Rapid Amplification of cDNA Ends (RACE)

Total RNA was extracted from *H. ventricosa* plant leaves using the RNAiso Plus reagent (TaKaRa, Tokyo, Japan). Reverse transcription (RT) was performed using an M-MLV enzyme (TaKaRa, Tokyo, Japan) and reverse primers listed in the Supplementary Table S1 followed by PCR-amplifications using a 2× Phanta DNA polymerase (Vazyme, Nanjing, China). The 3′ and 5′ end sequences of the novel virus genome were determined utilizing the HiScript-TS 5′/3′ RACE Kit (Vazyme, Nanjing, China) as instructed. The resulting products were gel-purified and subjected to Sanger sequencing. Based on the assembled sequence, the full-length sequence of viral genome was RT-PCR-amplified directly from the total RNA samples and Sanger sequenced.

### 2.4. Analyses of Viral Genome Sequence, Organization, and Phylogeny

ORFs in the novel viral genomic sequence were predicted using the ORF finder service (http://www.ncbi.nlm.nih.gov/orffinder, accessed on 10 December 2021) and the genomic sequence of the apple-associated luteovirus (AaLV, NC_040549) was utilized as the model. Amino acid (aa) sequences of the predicted viral proteins were then subjected to BLASTp search against the GenBank database. Domains in these predicted proteins were identified using the Conserved Domain Search Service (CD-Search) at the NCBI (https://www.ncbi.nlm.nih.gov/Structure/cdd/wrpsb.cgi, accessed on 10 January 2022). The sequence of the novel virus genome and its predicted viral proteins were aligned with the sequences of the known luteoviruses deposited in the GenBank database using the DNASTAR7.1 software (https://www.dnastar.com/, accessed on 22 February 2022). Detailed information about the downloaded sequences is provided in Supplementary Table S2.

To determine the phylogenetic relationship between this novel virus and other luteoviruses, the RdRp aa sequence of the novel virus was aligned with 25 representative viruses from different genera of the Tombusviridae using the ClustalW in the MEGA X software (version 11.0.11) (https://www.megasoftware.net/) and the phylogenetic analysis was performed utilizing the neighbor-joining method in the MEGA X software (version 11.0.11) with 1000 bootstrap replicates (https://www.megasoftware.net/). Detailed information about the downloaded sequences is provided in Supplementary Table S3.

### 2.5. Plasmid Construction and Agroinfiltration Assay

The predicted ORFs of the novel virus were individually RT-PCR-amplified from a total RNA sample prepared from an infected *H. ventricosa* leaf sample and cloned into the expression vector BGV005-HA, pCambia 1300-GFP, pGR106 (PVX-based constructs) or pCB301-RZ using the One-Step Cloning kit (Vazyme) as instructed. The resulting recombinant vectors were individually confirmed through Sanger sequencing and then transformed into *Agrobacterium tumefaciens* strain EHA105 or GV3101 cells through electroporation. The transformed agrobacteria were propagated overnight as described [20], diluted in an infiltration buffer (10 mM MgCl_2_, 10 mM MES, pH 5.8, and 100 μM acetosyringone) until OD600 = 0.8, and then individually infiltrated into the leaves of *N. benthamiana* plants with needleless syringes.

### 2.6. Western Blot Assays

Total protein was extracted from agroinfiltrated *N. benthamiana* leaves using an extraction buffer (50 mM Tris-HCl, pH 6.8, containing 9 M carbamide, 4.5% SDS, and 7.5% 2-mercaptoethanol). The resulting protein samples were separated in 12.5% sodium dodecyl sulfate-polyacrylamide gel electrophoresis (SDS-PAGE), transferred onto nitrocellulose membranes, and then analyzed using specific antibodies as previously described [22].

### 2.7. Confocal Microscopy and Transmission Electron Microscopy

The agroinfiltrated *N. benthamiana* leaves were detached at 48 h post agroinfiltration and examined under an FV3000 confocal laser scanning microscope (Olympus, Tokyo, Japan). RFP and GFP were excited at 561 nm and 488 nm, with emissions captured at 583 nm and 510 nm, respectively. The H2B-RFP fusion protein marks nuclei (as a nuclear marker), and GFP visualizes the subcellular localization of HVLV-encoded proteins. For the virion observation, the assayed *H. ventricosa* leaves were homogenized and the resulting homogenate was adsorbed onto formvar-coated 200-mesh copper grids before being negatively stained with 1% uranyl acetate. Virions were then observed and photographed using a transmission electron microscope (H-7650, Hitachi, Tokyo, Japan).

### 2.8. Construction and Validation of an Infectious cDNA Clone of HVLV

The full-length cDNA of HVLV was inserted into the binary vector pCB301-RZ using a two-fragment ligation strategy. The 5′ viral genomic fragment (nt 1–3015) and the 3′ viral genomic fragment (nt 3016–5723) were amplified by PCR using the primer pairs pCB301-HVLV-1-F/R and pCB301-HVLV-2-F/R, respectively (Supplementary Table S1). The resulting two fragments were sequentially inserted into the binary vector pCB301-RZ using the MultiS One-Step Cloning Kit (Vazyme, Nanjing, China). The resulting construct pCB301-HVLV was verified by Sanger sequencing. For agroinfiltration, the construct was transformed into *A. tumefaciens* strain GV3101. For RT-PCR detection of HVLV in inoculated plants, a primer pair (HVLV-detection-F/R) targeting the ORF3 (capsid protein) region was used to amplify a 606 bp product.

## 3. Results

### 3.1. Identification of a Novel Luteovirus in Hosta ventricosa

**Figure 1:**
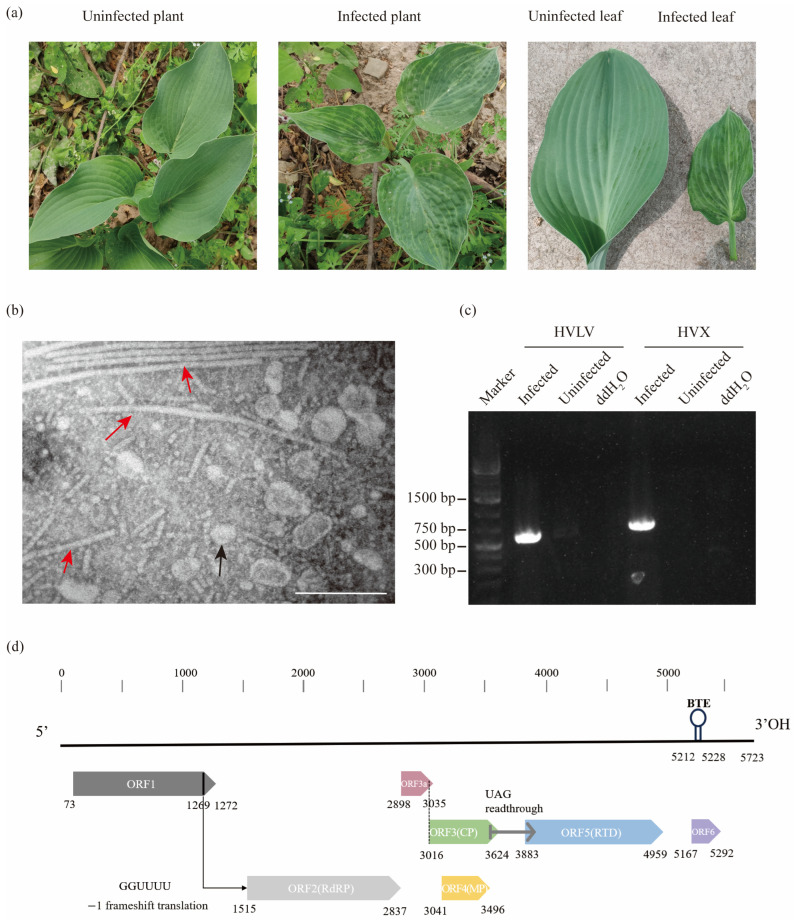
Symptoms of the analyzed *Hosta ventricosa* plant, virion morphology, and genome organization of HVLV. (**a**) Symptoms of an analyzed *Hosta ventricosa* plant. This diseased plant showed leaf chlorosis, mottle, mosaic and crinkling symptoms. (**b**) A TEM micrograph showing the morphology of HVLV and HVX virions in a leaf homogenate from the infected plant in (**a**). Red arrow indicates HVX virion, and black arrow indicates HVLV virion. Scale bar = 200 nm. (**c**) RT-PCR results showing HVLV and HVX co-infection in the infected *H. ventricosa* plant in (**a**) using virus-specific primer pairs. (**d**) Schematic diagram of HVLV genome organization. RdRP, RNA-dependent RNA polymerase; CP, coat protein; MP, movement protein; BTE, BYDV-like translational element. Nucleotide position of individual ORF is indicated.

The *H. ventricosa* plant showed leaf chlorosis, mottle, mosaic and crinkling symptoms (Figure 1a) and was collected for virus identification through RNA-seq. After filtering the low-quality reads, the resulting 24,866,033 clean reads were de novo assembled. BLAST searches of these assembled contigs identified two viral contigs, a 5716-nt contig (contig 1) and a 6536-nt contig (contig 2). An RdRp ORF was identified through a BLASTx search in the GenBank database using contig 1 or contig 2 as a query, exhibiting 45.17% aa sequence identity to that of rose spring dwarf-associated virus (RSDaV) (Table 1), and 99.39% aa sequence identity to that of HVX, respectively. Therefore, we concluded that this assayed *H. ventricosa* plant was co-infected with a novel virus belonging to the genus *Luteovirus* and HVX. We then examined the leaf extracts from the assayed *H. ventricosa* plant through transmission electron microscopy (TEM) and observed numerous flexuous filamentous virons (470–580 nm in length) resembling those of potexviruses and a few of spherical virions resembling those of luteoviruses (Figure 1b). These spherical virions range from 25 to 30 nm in diameter, which is consistent with the size of typical luteovirus particles. Next, we performed RT-PCR assays to determine the presence of these two viruses in the assayed *H. ventricosa* plant using the primers designed according to the contig 1 and 2. The RT-PCR results further confirmed this assayed *H. ventricosa* plant was indeed co-infected with this novel luteovirus and HVX (Figure 1c). To obtain the full-length genomic sequence of the novel luteovirus, we performed 5′ and 3′ end RACE followed by Sanger sequencing. The result revealed that the full-length genome of the novel luteovirus comprises 5723 nt (accession number PV547510; Figure 1d). Based on the findings above, we tentatively named this novel virus hosta ventricosa luteovirus (HVLV).

Further analysis of the HVLV genome identified seven open reading frames (ORFs), and 5′ and 3′ untranslated regions (UTRs) of 72 nt and 431 nt, respectively. The ORF1 (nt position 73–1272) was deduced to encode a 42.5 kDa P1 protein that shares the highest aa sequence identity (18.99%) with that of RSDaV. The ORF2 (nt position 1515–2837) was predicted to encode a 50.3 kDa P2 protein with a conserved luteovirus RdRp domain (cd23233, nt position 1662–2819) that shares the highest aa sequence identity (53.11%) with that of cherry-associated luteovirus (ChALV). The ORF1 also contained a −1 ribosomal frameshift sequence of GG**U**UUUU (nt position 1263–1269), which differs from the typical GG**K**UUUU motif in other luteoviruses. Additionally, the ORF1 and ORF2 were predicted to encode a P1–2 fusion protein with a molecular weight of 104.3 kDa through the −1 frameshift mechanism, which functions as the viral RdRP and shares the highest aa sequence identity of 45.17% with that of RSDaV. The ORF3a (nt position 2898–3035) was predicted to encode a 5.0 kDa long-distance movement-associated protein P3a using a non-AUG codon (AUA) for initiation, which shared the highest aa sequence identity of 51.79% with that of cereal yellow dwarf virus-RPV (CYDV-RPV). The ORF3 (nt position 3016–3624) was predicted to encode a 22.5 kDa capsid protein (CP) that shares the highest aa sequence identity (61.09%) with that of CYDV-RPV. The ORF4 (nt position 3041–3496) encodes a 17.0 kDa movement protein (MP) that contains a luteo_Vpg domain (cl03297) and shares the highest aa sequence identity (45.31%) with a P4 protein of CYDV-RPV. The ORF5 (nt position 3883–4959) was predicted to encode a 40.0 kDa protein P5 that has a PLrV (putative luteovirus-related protein V) ORF5 domain (pfam01690) and shares the highest aa sequence identity (27.31%) with that of bean leafroll virus (BLRV). In addition, an 80.7 kDa readthrough protein P3–5 was encoded via in-frame readthrough of the stop codon UAG of ORF3 [6]. The P3–5 readthrough protein shares the highest aa sequence identity (39.11%) with that of CYDV-RPV. HVLV genome was also found to contain an ORF6 (nt position 5167–5292), which was predicted to encode a 4.5 kDa protein P6 with an unknown function. This protein shares 4.17–7.29% aa sequence identity with those of other luteoviruses (Figure 1d; Table 1). Moreover, a barley yellow dwarf virus (BYDV)-like translational element (BTE, GGAUCCUGGGAAACAGG, nt position 5212–5228) was also found in the ORF6 of HVLV. The detailed information on each ORF, including nucleotide positions, encoded proteins, molecular masses, acquisition mechanisms, and predicted functions, is summarized in Table 2. Based on the current ICTV classification criteria for the genus *Luteovirus*, i.e., ≥10% aa sequence divergence in any viral proteins [6]; we conclude that HVLV represents a new species in the genus *Luteovirus*.

### 3.2. Phylogenetic Analysis of HVLV

**Figure 2:**
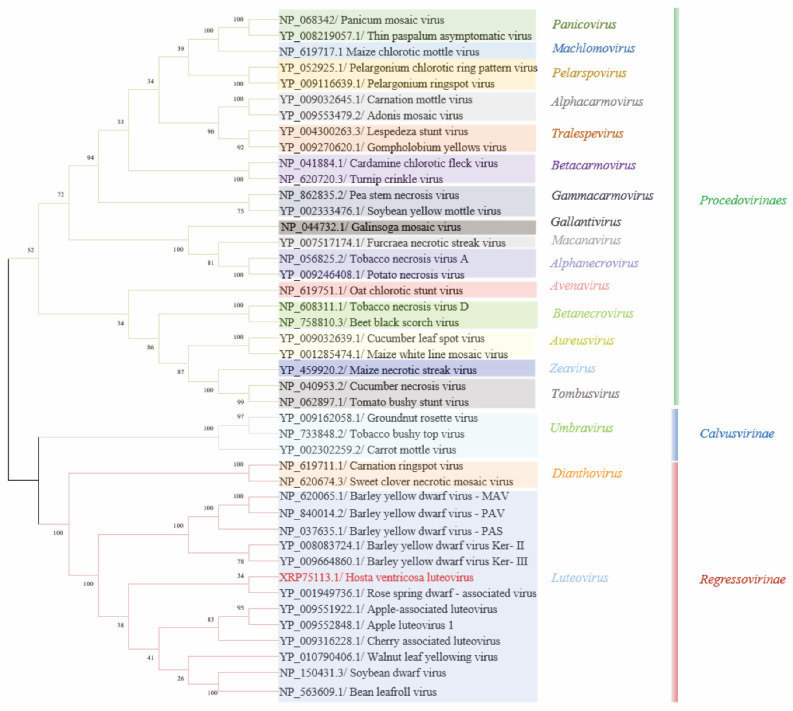
Phylogenetic relationships between HVLV and 43 representative members in different genera within the family *Tombusviridae*. The phylogenetic tree was generated using the RdRp aa sequences and the neighbor-joining method with 1000 bootstraps. The bootstrap values are indicated adjacent to the nodes. Viruses in the same genus are shown in the same color. HVLV is shown in red.

To elucidate the evolutionary relationship between HVLV and other representative known viruses in the *Tombusviridae* family, we constructed a phylogenetic tree based on RdRp aa sequences of HVLV and 25 representative members in 15 different genera in the subfamily *Procedovirinae*, three members in the *Umbravirus* genus in the subfamily *Calvusvirinae*, and 15 members in the *Dianthovirus* and *Luteovirus* genera in the subfamily *Regressovirinae* (Supplementary Table S3). The resulting phylogenetic tree showed that HVLV was clustered together with known viruses from the genus *Luteovirus* with most closely related to RSDaV (Figure 2), further indicating that HVLV is a novel species within the genus *Luteovirus*.

### 3.3. Subcellular Localizations of HVLV-Encoded Viral Proteins

**Figure 3:**
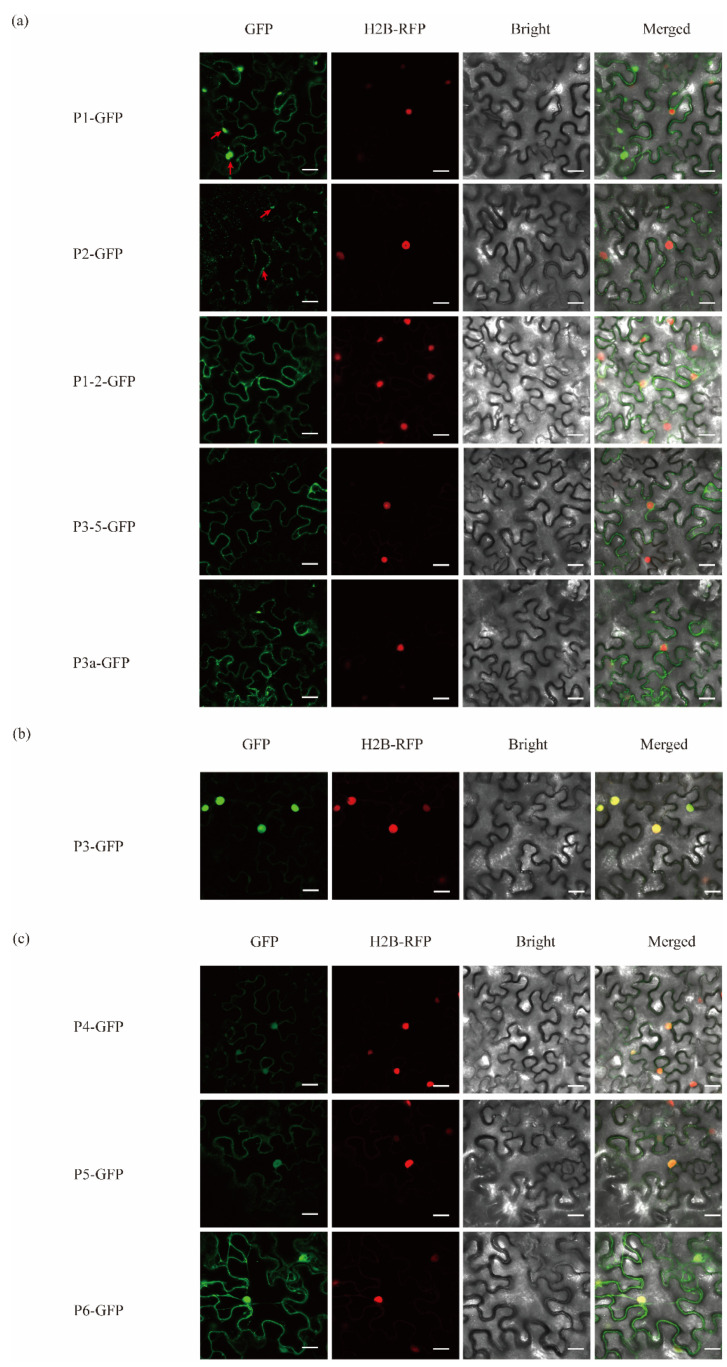
Subcellular localizations of HVLV-encoded viral proteins fused to GFP in *H2B-RFP* transgenic *N. benthamiana* leaf cells. Red fluorescence signals indicate nuclei. Scale bars = 20 μm. (**a**) Cytoplasmic localization of P1-GFP, P2-GFP, P1–2-GFP, P3–5-GFP and P3a-GFP. P1-GFP also formed large cytoplasmic aggregates, and P2-GFP also showed small punctate foci along the plasma membrane (arrows indicate representative signals for P1 and P2). (**b**) P3-GFP is exclusively localized in the nucleus. (**c**) P4-GFP, P5-GFP, and P6-GFP localize to both the nucleus and the cytoplasm.

To investigate biological functions of HVLV-encoded proteins, we first subcloned full-length *P1*, *P2*, *P3*, *P4*, *P5*, *P6*, *P1–2*, *P3–5* and *P3a* genes individually into the expression vector pCambia 1300-GFP to produce pC1300-P1-GFP, pC1300-P2-GFP, pC1300-P3-GFP, pC1300-P4-GFP, pC1300-P5-GFP, pC1300-P6-GFP, pC1300-P1–2-GFP, pC1300-P3–5-GFP, and pC1300-P3a-GFP. Agrobacteria harboring these expression vectors were individually infiltrated into *H2B-RFP* transgenic *N. benthamiana* leaves. Two days post-infiltration (dpi), the agroinfiltrated leaves were harvested and examined under a confocal microscope. The results showed that P1-GFP, P2-GFP, P1–2-GFP, P3–5-GFP, and P3a-GFP all localized to the cytoplasm (Figure 3a). Among them, P1-GFP forms large aggregates in the cytoplasm, and P2-GFP exhibits smaller punctate foci along the plasma membrane (Figure 3a). The P3-GFP fusion was exclusively localized to the nucleus (Figure 3b), while all P4-GFP, P5-GFP, and P6-GFP fusions were localized to both the nucleus and the cytoplasm (Figure 3c).

### 3.4. Pathogenicity of HVLV-Encoded Viral Proteins

**Figure 4:**
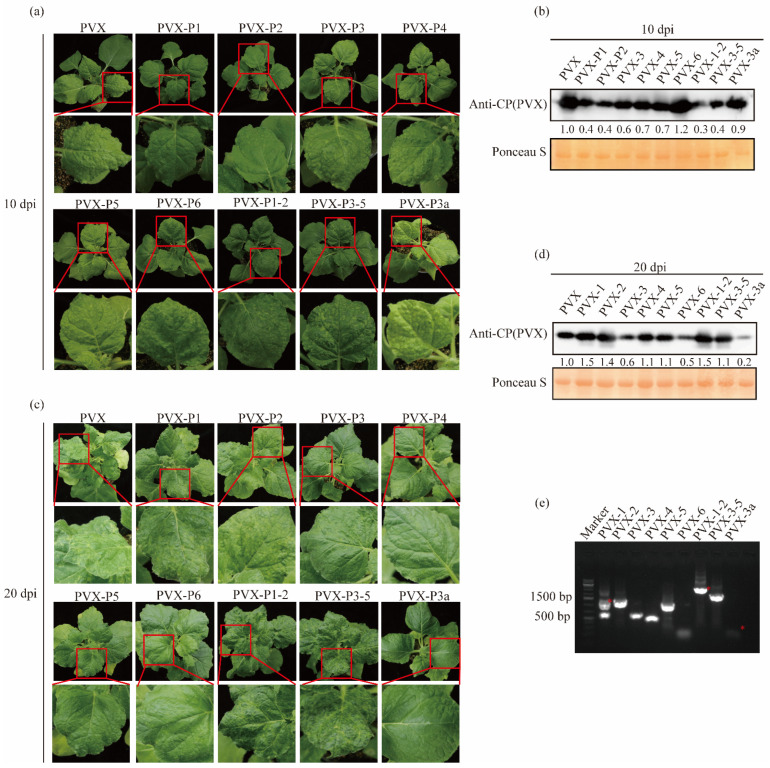
Symptom determinant identification of HVLV using PVX heterologous expression system. (**a**) Symptoms of *N. benthamiana* plants inoculated with PVX-P1, PVX-P2, PVX-P3, PVX-P4, PVX-P5, PVX-P6, PVX-P1–2, PVX-P3–5 or PVX-P3a at 10 dpi. Magnified leaf areas (insets) are shown to better visualize symptom phenotypes. The experiment was performed in three biological replicates with similar results, and the representative image is shown. (**b**) Western blot assay was used to analyze the accumulation of PVX CP in systemic leaves of the inoculated *N. benthamiana* at 10 dpi. The Ponceau S-stained large RuBisCO subunit band was used to show sample loading. The intensity of protein bands was quantified using the ImageJ software (version 1.8.0). (**c**) Symptoms of *N. benthamiana* plants inoculated with PVX-P1, PVX-P2, PVX-P3, PVX-P4, PVX-P5, PVX-P6, PVX-P1–2, PVX-P3–5 or PVX-P3a at 20 dpi. Magnified leaf areas are shown to better visualize symptoms. The experiment was repeated three times with similar results, and the representative image is shown. (**d**) Western blot assay was used to analyze the accumulation of PVX CP in systemic leaves of the inoculated *N. benthamiana* leaves at 20 dpi. The Ponceau S-stained large RuBisCO subunit band was used to show sample loading. The intensity of protein bands was quantified using the ImageJ software (version 1.8.0). (**e**) RT-PCR detecting *P1*, *P2*, *P3*, *P4*, *P5*, *P6*, *P1–2*, *P3–5* and *P3a* genes in recombinant PVX-inoculated *N. benthamiana* plants at 20 dpi. The red asterisks indicate the correct amplicon.

A potato virus X (PVX)-based vector was used to transiently express HVLV-encoded viral proteins in *N. benthamiana* plants as described previously [23]. To do this, each HVLV protein gene was amplified and inserted into the PVX-based vector (pGR106) downstream of the coat protein (CP) ORF to generate constructs expressing P1 to P6, P1–2, P3–5, and P3a, respectively. For simplicity, the PVX-based constructs expressing HVLV proteins are designated as PVX-P1 to -P6, PVX-P1–2, PVX-P3–5, and PVX-P3a (e.g., pGR106-HVLV-P1 corresponds to PVX-P1). Then, these constructs were separately transformed into *A. tumefaciens* strain GV3101 cells and then infiltrated into *N. benthamiana* leaves. The *N. benthamiana* plants infiltrated with the original PVX infectious clone were used as the control. At 10 days post-infiltration (dpi), plants infected with PVX-P5 and PVX-P3a exhibited leaf yellowing symptoms distinct from the mosaic symptoms induced by PVX infection. This phenotype aligns with the typical symptoms associated with infections by viruses of the genus *Luteovirus*, suggesting that P5 and P3a may contribute to the yellowing symptoms observed in HVLV and HVX co-infected plants. Meanwhile, at 10 dpi, plants infected with PVX-P1–2 and PVX-P3–5 displayed leaf mottling that differed from the mosaic symptoms caused by PVX, implying that P1–2 and P3–5 may be associated with the mottling symptoms resulting from HVLV infection. Furthermore, none of the experimental groups showed significant differences in the mosaic symptoms caused by PVX compared to the PVX control group (Figure 4a). At 20 dpi, the plants infected with PVX-P1 or PVX-P2 exhibited similar mosaic symptoms to those caused by PVX infection, while the plants infected with PVX-P3, PVX-P4, PVX-P5, PVX-P6 or PVX-P3a exhibited the recovery of disease symptoms. In contrast, the PVX-P1–2- or PVX-P3–5-infected plants developed much stronger mosaic symptoms than the PVX-infected control plants (Figure 4c).

To determine which viral protein(s) could regulate PVX replication in the infected plants, we analyzed the accumulation level of PVX CP through Western blot assay using a PVX CP-specific antibody. The result showed that at 10 dpi, the accumulation level of PVX CP was reduced in all recombinant PVX-infected plants compared to the PVX-infected control plants, except those infected by the PVX-P6 (Figure 4b). At 20 dpi, the accumulation level of PVX CP in the PVX-P1-, PVX-P2-, PVX-P1–2- or PVX-P3–5-infected plants were slightly higher than that in the PVX-infected control, while the accumulation level of PVX CP was lower in the PVX-P3-, PVX-P6- or PVX-P3a-infected plants than that in the PVX-infected control. In addition, the accumulation level of PVX CP in the PVX-P4- or PVX-P5-infected plants was comparable to that in the PVX-infected control (Figure 4d). RT-PCR detection results confirmed that *P1*, *P2*, *P3*, *P4*, *P5*, *P6*, *P1–2*, *P3–5* and *P3a* genes were in recombinant PVX-inoculated *N. benthamiana* plants at 20 dpi (Figure 4e). Based on the above findings, we conclude that both P1–2 and P3–5 are viral pathogenic determinants.

### 3.5. RNA Silencing Suppressors of HVLV-Encoded Viral Proteins

**Figure 5:**
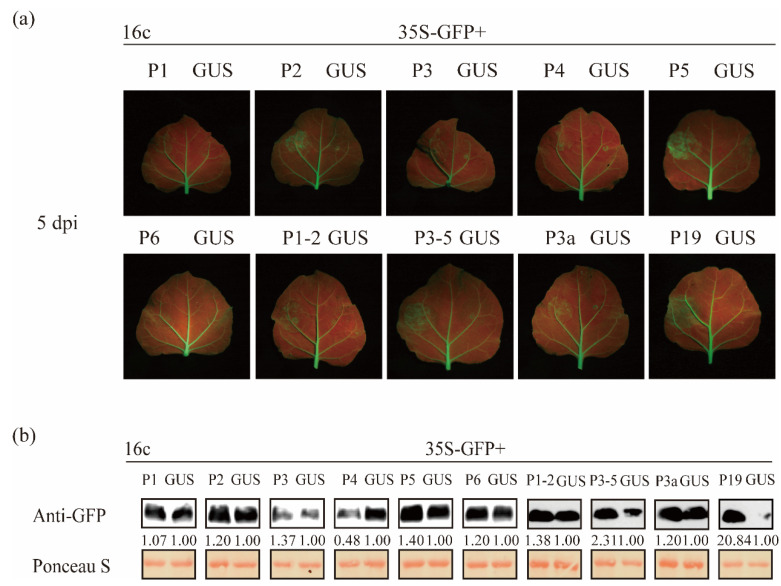
HVLV-encoded P2, P5, and P3–5 proteins all function as RNA silencing suppressors. (**a**) The leaves of 16c *N. benthamiana* plants were co-infiltrated with two constructs expressing GFP and P1-HA, P2-HA, P3-HA, P4-HA, P5-HA, P6-HA, P1–2-HA, P3–5-HA, P3a-HA, P19 (positive control), or GUS-HA (negative control). The infiltrated leaves were photographed under UV light at 5 dpi. The experiment was repeated three times with similar results. (**b**) Western blot assay was used to analyze GFP accumulation in the leaves shown in figure (**a**) above. The Ponceau S-stained large RuBisCO subunit band was used to show sample loading. The intensity of protein bands was quantified using the ImageJ software (version 11.0.11).

RNA interference (RNAi) is a critical antiviral immune defense mechanism in plants. However, plant viruses have evolved to encode RNA silencing suppressors to counteract this plant immunity [27,28]. In the GFP-based RNA silencing assay using 16c transgenic *N. benthamiana*, the transcript of exogenous *GFP* triggers the RNA silencing of the endogenous *GFP* gene, which reduces or abolishes GFP fluorescence in infiltrated leaves. A viral protein with silencing suppressor activity can block this RNA silencing, thereby preserving GFP fluorescence in infiltrated leaves. The GFP fluorescence was observed under UV light and the GFP accumulation was determined by Western blot. To identify viral protein(s) possessing RNA silencing suppressor activity, we separately cloned viral protein genes of HVLV into the expression vector BGV005-HA. An agrobacterium culture carrying a plasmid expressing the single-stranded GFP RNA (ssGFP) was mixed at a 1:1 ratio with an agrobacterium culture carrying a plasmid expressing P1-HA, P2-HA, P3-HA, P4-HA, P5-HA, P6-HA, P1–2-HA, P3–5-HA or P3a-HA; then, it was co-infiltrated into the leaves of 16c transgenic *N. benthamiana* plants. All agroinfiltrations were performed in 1-cm^2^ parallel symmetric regions of fully expanded *N. benthamiana* leaves. The mixed agrobacterium cultures expressing ssGFP and P19 or ssGFP and GUS-HA were used as the positive and negative controls. At 5 dpi, the leaves were harvested and examined under a UV lamp. The leaves co-expressing ssGFP with P2-HA, P5-HA or P3–5-HA showed clear GFP green fluorescence similar to the positive control (ssGFP + P19), In contrast, GFP fluorescence was suppressed in leaves co-expressing ssGFP with P1-HA, P3-HA, P4-HA, P6-HA, P1–2-HA, or P3a-HA, resembling the negative control (ssGFP + GUS-HA) (Figure 5a). Western blot assay using a GFP-specific antibody further revealed that the accumulation level of GFP in the leaves co-expressing ssGFP + P5-HA, ssGFP + P3–5-HA or ssGFP + P19 was higher than that in the leaves co-expressing ssGFP + GUS-HA (Figure 5b). Although the Western blot signal for P2 was weaker than expected (Figure 5b), likely due to technical limitations in protein extraction or transfer, the consistent UV fluorescence across replicates supports the conclusion that P2 exhibits RSS activity. Collectively, these findings indicate that P2, P5 and P3–5 proteins of HVLV are all viral RNA silencing suppressors.

### 3.6. Infectivity of an Infectious cDNA Clone of HVLV

**Figure 6:**
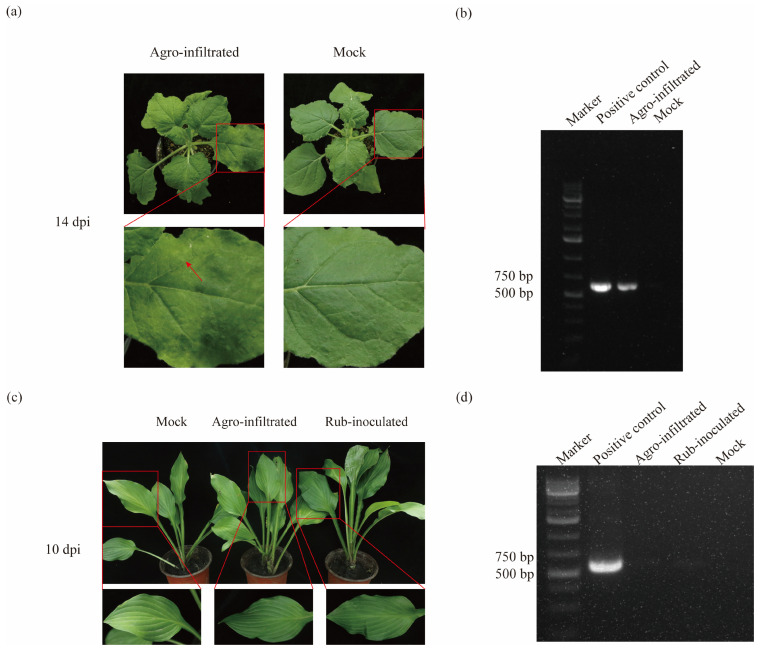
Agroinfiltration inoculation of the infectious cDNA clone of HVLV in *N. benthamiana* plants. (**a**) Symptoms of *N. benthamiana* plants inoculated with pCB301-HVLV or empty pCB301 vector (Mock) through agroinfiltration. The inoculated plants were photographed at 14 dpi. Arrows indicate leaf chlorosis and mottle symptoms. (**b**) RT-PCR detection of HVLV in the inoculated plants in figure (**a**) using a HVLV-specific primer pair. (**c**) Symptoms of *H. ventricosa* plants at 10 dpi after agroinfiltration with pCB301-HVLV or rub inoculation with leaf extracts from HVLV-infected *N. benthamiana* plants. (**d**) RT-PCR detection of HVLV in the systemic leaves of the inoculated *H. ventricosa* plants shown in (**c**) using the HVLV-specific primer pair.

The construction of viral infectious clone is indispensable for studying viral biological characteristics and gene functions. We constructed a full-length infectious cDNA clone of HVLV using the pCB301-RZ vector as the backbone. The resulting infectious clone pCB301-HVLV then was inoculated into *N. benthamiana* leaves through agroinfiltration, and the inoculated *N. benthamiana* plants exhibited leaf chlorosis and yellowing symptoms at 14 dpi (Figure 6a). RT-PCR analysis of systemic leaves collected from the inoculated *N. benthamiana* plants using a HVLV-specific primer pair targeting the ORF3 region displayed that these assayed plants were indeed systemically infected with HVLV (Figure 6b), indicating that an infectious cDNA clone of HVLV was successfully constructed, and HVLV can infect *N. benthamiana* plants.

To determine whether the HVLV infectious clone can also infect its natural host *H. ventricosa*, we performed agroinfiltration of pCB301-HVLV into *H. ventricosa* leaves. In addition, rub inoculation of *H. ventricosa* plants was carried out using leaf extracts from HVLV-infected *N. benthamiana* plants. However, neither agroinfiltration nor rub inoculation resulted in the establishment of systemic infection in *H. ventricosa* plants. At 10 dpi, no visible symptoms were observed on systemic leaves (Figure 6c), and RT-PCR using the HVLV-specific primer pair targeting the ORF3 region failed to detect viral RNA in these tissues (Figure 6d).

## 4. Discussion

The genus *Luteovirus* has recently been categorized into the family *Tombusviridae*. Viruses in this genus have been reported to infect various plant hosts, including poaceous, fabaceous, solanaceous, brassicaceous, asteraceous, caryophyllaceous, and rosaceous woody species [29]. In this work, a *H. ventricosa* plant with leaf chlorosis, mottle and crinkling symptoms was investigated using RNA-seq, TEM, RT-PCR, and DNA sequencing, and it was discovered that this assayed plant was co-infected with HVX and a novel luteovirus. Based on the aa sequence identities of all predicted viral proteins (all below 62.0%, Table 1), the close phylogenetic relationship with the members in the genus *Luteovirus*, and the ICTV species demarcation criteria for luteoviruses [6], we propose that this novel virus represents a new species tentatively designated as hosta ventricosa luteovirus (HVLV) within the genus *Luteovirus*. The genome of HVLV is a 5723 nt positive-sense, single-stranded RNA. Similar to known luteoviruses, the HVLV genome contains six ORFs, encoding the replication-associated P1 and P1–2 fusion proteins, CP, movement-associated P3a and P4 proteins, and aphid transmission-associated P3–5 fusion protein. Furthermore, the HVLV genome also contains ORF6 to encode P6 with an unknown function. It also contains a 17 nt sequence (GGAUCCUGGGAAACAGG) that forms the BYDV-like translational element [9,12,13,30]. Both P1–2 and P3–5 function as potential viral pathogenicity determinants in the PVX-based heterologous expression system in *N. benthamiana* plants (Figure 4).

In the PVX-based heterologous expression system, the PVX CP accumulation differences between 10 and 20 dpi likely arise from the effect of HVLV viral proteins on PVX replication or/and the progressive activation of host RNA silencing. HVLV proteins with RSS activity (P2, P3–5) may strongly suppress host RNA silencing at a later stage, leading to higher PVX accumulation, whereas other viral proteins (e.g., P3, P3a) may interfere with PVX replication, leading to lower PVX accumulation. This phenomenon has been shown in a previous report [23]. The divergent behaviors of different HVLV proteins highlight the complexity of virus–host interactions.

RNAi is a highly conserved and important defense mechanism in eukaryotic cells to resist viral invasion [31]. During long-term evolution, plant viruses have evolved to encode RNA silencing suppressors (RSS) to counteract the host RNAi defense during their infections [32]. For example, the tomato bushy stunt virus-encoded p19 protein and tobacco etch potyvirus-encoded HC-Pro protein are known to bind siRNAs to disrupt the Dicer enzyme and RNA-induced silencing complex (RISC) activity [33]. In 2013, Shen and others reported BYDV P4, the first identified RSS in luteoviruses, functions by targeting the Argonaute (AGO) protein in the nucleus [34]. In this study, the P2, P5, and P3–5 proteins of HVLV have been found to exhibit RNA silencing suppressor activity in the 16c *N. benthamiana* assay system, but not the P4 protein, suggesting functional divergence (Figure 5). Which RNAi step(s) is targeted by P2, P5, and P3–5 proteins is currently unclear and requires further investigation.

Luteoviruses are phloem-limited viruses and are transmitted by aphids in a circulative and non-propagative manner [35]. The CP proteins of luteoviruses are encoded by the ORF3, and the P3–5 protein is translated via readthrough of the ORF3 stop codon. In this study, the HVLV P3–5 protein has been found to function as a potential viral pathogenicity determinant through a heterologous expression system using a PVX-based vector. This finding supports earlier reports that the luteovirus P3–5 protein is involved in both aphid transmission and viral accumulation in infected plants [36,37]. Although the CPs of luteoviruses are known to determine the specificity of insect transmission, the insect vector of HVLV has not been determined in this study.

In this work, an infectious cDNA clone of HVLV has been constructed. Inoculation of this infectious clone to *N. benthamiana* plants through agroinfiltration caused leaf chlorosis and yellowing symptoms. This infectious clone provides an essential tool for future studies on viral gene function and pathogenicity. However, agroinfiltration of this infectious clone into *H. ventricosa* plants failed to establish local and systemic infection in inoculated plants. Additionally, rub inoculation of *H. ventricosa* plants using HVLV-infected *N. benthamiana* leaf extracts also failed (Figure 6). The successful infection of *N. benthamiana* by the HVLV infectious clone indicates host compatibility, whereas the failure infection in *H. ventricosa* by the HVLV infectious clone or rub inoculation suggests that HVLV requires its vector for its field transmission. Thus, we speculate that HVLV may be transmitted between natural host plants by a certain vector. The co-occurrence of HVLV and HVX in the single analyzed plant indicates that both viruses can co-infect *Hosta ventricosa* plants. Whether they share a common insect vector remains unknown and requires future field surveys and vector transmission experiments.

It is important to note that the field *H. ventricosa* plants analyzed in this study were co-infected with HVX and HVLV. The observed symptoms (leaf chlorosis, mottle and crinkling) in the analyzed field plant were more severe than those typically associated with HVX alone in other Hosta species [38]. While HVX usually causes chlorosis and mosaic symptoms or no visible symptoms on tolerant varieties [4], the additional and intense chlorosis and crinkling symptoms may result from synergistic interactions between HVLV and HVX. Furthermore, we used a PVX-based heterologous expression system to assess the pathogenicity of individual HVLV proteins and discovered that both viral P1–2 and P3–5 contribute to its pathogenicity (Figure 4). However, under natural conditions, the specific contribution of HVLV to symptom development remains to be further elucidated in our next study. Our conclusion is based on a single field plant; therefore, larger field surveys are needed to confirm the generality of these findings. The co-occurrence of HVX (a member of the family *Alphaflexiviridae*) and HVLV (a member of the family *Tombusviridae*) highlights the complexity of viral communities in plants, where multiple pathogens may interact to exacerbate disease.

## 5. Conclusions

In conclusion, we have identified a novel luteovirus tentatively named hosta ventricosa luteovirus (HVLV) and HVX in the analyzed *H. ventricosa* plant. HVLV belongs to the genus *Luteovirus*, within the family *Tombusviridae*, and its genome comprises 5723 nucleotides and seven ORFs. HVLV-encoded P1–2 and P3–5 proteins all function as potential viral pathogenicity determinants, and the P2, P5 and P3–5 proteins exhibit RSS activities. HVLV can infect *N. benthamiana* plants through inoculation of an infectious clone and cause systemic leaf chlorosis and yellowing symptoms. These findings benefit our understanding of luteoviruses and the viruses of *H. ventricosa* and also provide useful information for the development of effective control strategies for *H. ventricosa* viral diseases.

## Figures and Tables

**Table 1:** Viral protein amino acid sequence identities between HVLV and other known luteoviruses.

Virus Names	Amino Acids Identities (%)								
	RdRp	P1	P2	P3a	P3	P4	P3–5	P5	P6
Apple luteovirus 1	41.95	18.00	47.60	48.21	34.84	19.79	22.48	15.40	4.17
Apple-associated luteovirus	43.51	18.28	52.93	50.00	36.20	18.23	21.55	15.40	4.17
Barley yellow dwarf virus-PAV	40.29	15.47	51.69	32.14	39.37	22.40	23.30	16.02	6.25
Barley yellow dwarf virus-MAV	40.29	14.77	52.40	30.36	36.20	20.83	22.72	16.63	6.25
Barley yellow dwarf virus-PAS	39.93	14.91	51.87	32.14	38.46	21.35	22.95	16.63	5.21
Barley yellow dwarf virus Ker-II	40.29	15.19	51.15	33.93	37.56	20.31	21.19	14.17	2.08
Barley yellow dwarf virus Ker-III	39.37	13.78	51.51	None	35.29	20.83	21.78	12.73	None
Bean leafroll virus	43.42	17.86	52.58	39.29	41.63	25.52	31.97	27.31	None
Cereal yellow dwarf virus-RPV	9.75	4.36	9.77	51.79	61.09	45.31	39.11	26.49	None
Cherry-associated luteovirus	43.61	17.30	53.11	21.43	35.75	17.19	22.13	15.81	4.17
Walnut leaf yellowing virus	39.93	12.38	49.73	None	27.60	None	17.10	12.53	None
Red clover-associated luteovirus	44.53	17.72	52.93	None	26.24	None	16.98	12.53	7.29
Rose spring dwarf-associated virus	45.17	18.99	50.98	42.86	30.32	18.75	21.66	17.66	6.25
Soybean dwarf virus	43.70	17.72	46.71	None	47.51	33.85	31.15	22.18	None

Note: The highest aa sequence identities are shown in red. None, the viruses do not contain this ORF.

**Table 2:** ORF organization, encoded proteins, and predicted functions of HVLV.

ORF	Nucleotide Position	Encoded Protein (s)	Molecular Mass (kDa)	Acquisition Mechanism	Predicted Function
ORF1	73–1272	P1	42.5	Conventional translation	Unknown; replication-associated
ORF1 + ORF2	73–2837	P1–2 (fusion)	104.3	−1 ribosomal frameshift	RNA-dependent RNA polymerase (RdRp)
ORF2	1515–2837	P2	50.3	Conventional translation	Contains RdRp domain
ORF3a	2898–3035	P3a	5.0	Non-AUG initiation (AUA)	Long-distance movement
ORF3	3016–3624	P3 (CP)	22.5	Conventional translation	Coat protein
ORF4	3041–3496	P4 (MP)	17.0	Conventional translation	Cell-to-cell movement protein
ORF3 + ORF5	3016–4959	P3–5 (fusion)	80.7	Readthrough of ORF3 stop codon (UAG)	Aphid transmission
ORF5	3883–4959	P5	40.0	Conventional translation	Contains PLrV_ORF5 domain
ORF6	5167–5292	P6	4.5	Conventional translation	Unknown; contains BTE

## Data Availability

The complete genome sequence of HVLV has been deposited in the NCBI GenBank database under accession number PV547510. All other data generated or analyzed during this study are included in this published article and its Supplementary Materials.

## Supplementary Materials

The following supporting information can be downloaded at: https://www.mdpi.com/article/10.3390/v18070798/s1, Table S1: Primers applied in this study; Table S2: Viruses in the genus *Luteovirus* used for amino acid identity analyses of viral protein; Table S3: Viruses used for phylogenetic analysis.
